# Researches on the Chemistry of the Food

**Published:** 1847-10-01

**Authors:** 


I. Researches on the Chemistry of Food. By *Justus Liebig*, M.D. Edited from the Manuscript of the Author. By *William Gregory*, M.D. 8vo. pp. 156. Taylor and Walton, 1847.

II. The Chemistry of Vegetable and Animal Physiology. By Dr. *G. J. Mulder*. Translated from the Dutch by Dr. *P. F. H. Fromberg*, with an Introduction and Notes. By *James F. W. Johnston*, F.R.S. L. & E. Part III. 8vo. pp. 267. Eight coloured Lithographs. Blackwood, Edin. 1847.

Although these recent works, or rather portions of works, of the two celebrated rival chemists, deal too much in special details to admit of lengthened notice, valuable as these may prove for the purposes of reference, at least when some of the statements they contain have been corroborated by farther investigation, they also refer to matters of a more general character which may advantageously be adverted to here. While there is such discrepancy in important statements made as to the results of minute analyses by men who should be incontrovertible authorities upon subjects to which the whole energies of their lives have been devoted, we may be well excused detailing that portion of their labours, in the hopes that renewed investigation, patiently and charitably conducted, may reconcile differences.

Professor Liebig commences his Essay with an introductory chapter upon the " *Methods of Investigation in Animal Chemistry* ;" in which he endeavours to demonstrate that, if the active spirit of chemical research which has now been going on for the last ten years is so barren in actual and assured results, this has arisen principally from the faulty mode in which the investigations have been carried on, in consequence of which erroneous, insufficient and premature conclusions have been arrived at. As an example of these, Mulder's Proteine Theory is put prominently forward, in a tone of assumed superiority, not altogether becoming one who once acknowledged its validity, or very well calculated to heal breaches of good understanding already made.

One source of error arising from the isolation of the researches of the chemist from those of the physiologist and pathologist, is thus touched upon.

"In modern times this method (hypothetical systems of medicine) has been abandoned as entirely unproductive; but, on the other hand, men commit an error not less grave, inasmuch as, instead of acquiring by their own researches the knowledge necessary for the solution of their difficulties, they leave this duty to others, who, fully occupied with the cultivation of their own branch of science, have neither interest in the questions to be solved, nor inclination for the task. From the chemical analysis of blood, of urine, or of a morbid product, they expect an aid which these analyses can never afford, as long as the results of the chemist are not brought into the true connection with the conditions which they are to explain, or with the causes which have produced these conditions. All the new facts daily ascertained by the chemist are regarded by pathologists as being exactly those which are of no direct use to them, because they have no clear idea of that which they require; because they are unable to connect with these chemical discoveries any question to be solved, or to draw from them any conclusion.

"What an inconceivable delusion, what a confusion of ideas must exist, when a physician thinks, that from the complex results of an analysis of the blood, he can draw a conclusion as to the nature and the cause of a disease, and can found on this a method of treatment, when we have not yet advanced so far in physiology as to bring into relation with the digestive process one of the simplest chemical facts, namely, the absence of alkaline phosphates in the urine of the herbivora! What pathologist has ever yet attempted to fix and define the notion of bad or spoiled food, in its full signification, by means of a logical comparison with good and wholesome food? and yet the former are regarded as the proximate causes of diseased conditions. I really admit, that for such an investigation chemical knowledge is indispensable; but the investigation itself has no value in reference to chemistry, and constitutes no object of research for the chemist, as such.

"From this state of things, which depends on the want of connection between the labours of chemists and those of physiologists, it has happened, that Animal Chemistry, during the last ten years, has gained little more than a more accurate knowledge of those compounds which the animal organism applies to no further purpose in its economy; and that, at the present time, it seems as if all the wonderful properties which it exhibits were produced only by means of albumen, fibrine, gelatine, some cerebral or nervous matter, and a little bile. It is universally felt, that we are as far from a true animal chemistry as the anatomy of the last century was from the physiology of the present day. Indeed the animal chemistry of our time cannot be compared to modern anatomy, since microscopic researches have established the existence of structures which had entirely escaped the earlier investigators; of structures, as is now known, on which alone the function of those formerly observed depends." P. 5.

The knowledge derived from *ultimate Analysis* which has of late been so industriously pursued, is here shown to have been more apparent than real—the formulæ thus accumulated too often proving data of little worth.

"About ten years since, the ultimate analysis of organic bodies furnished physiology with a result highly important, in order to the easy understanding of the digestive or nutritive process, by demonstrating, that fibrine, albumen, and caseine have the same composition. Misled by this result, many chemists thought that the chief problem to be solved by chemistry was to ascertain, by ultimate analysis, the composition, in 100 parts, of all the constituents of the body; and thus many were induced to act on each of these constituents, without a more minute study of its chemical relations and its properties, with alcohol, ether, and acids; and with the aid of the known resources of organic analysis, to determine the percentage of carbon, nitrogen, hydrogen, and oxygen. They believed that they had thus, by means of these numerical results, done a real service to physiology, although the only addition thus made to the name of the substance analysed was an empty formula, of the accuracy of which there was no evidence whatever. Now that we have been for ten years in possession of these formulæ, every one must perceive that we have made no real progress. The cause of this is obvious to all who know the true value of ultimate analysis. Ultimate analysis is a means of acquiring knowledge, but is not itself that knowledge. Even supposing, what no one will seriously maintain with regard to the constituents of the animal body, that analysis had made us acquainted with the exact proportions in which their elements are united together, yet this knowledge gives us not the least information as to the arrangement of these elements, or the way in which they group themselves, under the influence of chemical agencies. Now it is the knowledge of both these things together which alone can lead us to definite views as to the part which these compounds play in the vital processes, or the changes to which they are subjected up to the period of their expulsion from the body; and this is essentially the problem which Chemistry has to solve in reference to the vital process.

"Ultimate analysis, by itself, has this peculiarity, that in the case of very complex substances it cannot secure the chemist against errors, because there is no other control for the accuracy of the analysis than the analysis itself; and because the errors are equal at different times, and escape notice when we cannot change the methods of determining the individual elements. Now there is as yet no means of determining the weight of carbon otherwise than in the form of carbonic acid, or that of hydrogen otherwise than in the form of water.

"The only way to attain an accurate expression for the composition of those substances, which, like the constituents of the animal body, contain a very large number of elementary molecules in the complex atom of the compound, is to endeavour to resolve it into two or more less complex compounds, and to compare the composition and the amount of these products with those of the body from which they have been derived." P. 12.

The main part of Liebig's work is occupied in detailing his analytical investigation of the *Constituents of the Juices of Flesh*. After alluding to the crude views formerly entertained respecting the presence of Lactic Acid, he dwells at some length upon the properties of *Kreatine* discovered by Chevreul in 1835 to be a peculiar crystallizable body obtainable by boiling flesh with water. Since then other chemists have obtained it in small quantities, while others failing to do this have considered its presence accidental. Liebig details the process by which it may be obtained, providing largish quantities are operated upon. From 8 to 10 lbs. of flesh must be employed, and even from this, after the coagulation of the albumen and colouring matter, soluble matters to the amount of 4 ozs. only will be obtained for investigation, and that but by the use of a good press. The fluid obtained possesses an acid re-action, and even in those animals in which it has only been obtained in conjunction with blood, the alkali of this does not neutralize the free acid of the flesh. "Indeed, I believe, that in most animals, if we suppose the whole mass of blood in the vessels to be mixed with the whole fluid of the muscles, the mixture would retain, not a neutral or alkaline, but an acid re-action." The free acid is removed by means of a solution of baryta, giving rise to precipitates of the phosphate of baryta and of magnesia. The filtered fluid is then gently evaporated until the crystals of Kreatine are deposited. The amount of this substance produced varies not only in the flesh of different kinds of animals, but even in animals of the same class—the quantity being always much less in fat flesh than in lean flesh. 1000 parts of the flesh of lean fowls yielded 3·05 of Kreatine: 1000 of the horse 0·72: 1000 of the ox 0·697. Obtained and purified Kreatine is neither acid or basic. When Kreatine is dissolved in heated strong hydrochloric, sulphuric, phosphoric, or nitric acid, and the solution evaporated, crystals are obtained which are very soluble in alcohol, which those of Kreatine are not. By this contact with the strong mineral acids a portion of these enters into combination with the Kreatine, and a new body, of totally different chemical properties, a true organic alkali, and termed by Liebig *Kreatinine* is produced. This is far more soluble than Kreatine; and in its chemical character is quite analogous to ammonia.

The compound discovered in the urine three years since by Pettenkoffer contains the same proportions of carbon and nitrogen as do Kreatine and Kreatinine; and a repetition of Pettenkoffer's analysis by Liebig shows this substance to be a mixture of Kreatine with a little Kreatinine: and although neither of these bodies exist in great amount in the urine, yet may they be more conveniently procured from it than from flesh.

After describing the Salts of Kreatinine, Liebig gives an account of another organic base termed by him *Sarcosine,* produced by adding to a boiling saturated solution of Kreatine ten times the weight of crystallized hydrate of baryta. Ammonia is disengaged and carbonate of baryta precipitated, while a colourless fluid containing Sarcosine and caustic baryta is produced.

"If from the elements of crystallised kreatine we subtract those of sarcosine, there remains a formula exactly identical with that of urea.

| | | |
|---|---|---|
| From 1 eq. Kreatine | = | $C_8\ N_3\ H_{11}\ O_6$ |
| Deduct 1 eq. Sarcosine | = | $C_6\ N_4\ H_7\ O_4$ |
| There remains 1 eq. Urea | = | $C_2\ N_2\ H_4\ O_2$ |

"It is consequently obvious that, in the decomposition of kreatine by baryta, carbonic acid and ammonia are secondary products derived from the decomposition of urea. I have ascertained that a solution of urea in barytic water is resolved by long boiling into carbonate of baryta and ammonia with the same appearances as those above described; and I have also ascertained that urea is present in the liquid when kreatine is boiled with baryta, if examined before the whole of the kreatine is decomposed." P. 75.

By the agency of alcohol upon the liquid from flesh, whence all the Kreatine has been deposited, crystals of an acid, termed by Liebig *Inosinic,* may be procured. After describing this and the compounds it forms with baryta, potash, &c., and detailing a simple process by which Kreatinine may be extracted from muscle, the author next describes *Lactic Acid* as forming one of the constituents of flesh, and passes on to the consideration of the *Inorganic Constituents of the Juices of Flesh*, which he treats of at very considerable length. Into his ingenious speculations upon the important agency of the saline constituents, the alkaline phosphates, of the juice of flesh, and upon the function of the phosphate of soda in the blood —that of absorbing carbonic acid and giving it out in the lungs—our limits forbid our following him. An extract or two will exhibit the interesting character of the subjects adverted to. After stating that *Lactic Acid* cannot be detected in the urine even of those to whom it has been administered, he goes on to observe.

"From this it plainly appears, that the lactic acid in the organism is employed to support the respiratory process, and the function performed by sugar, starch, and in general all those substances which, in contact with animal matter are convertible into lactic acid, ceases to be an hypothesis. These substances are converted in the blood into lactates, which are destroyed as fast as they are produced, and which only accumulate where the supply of oxygen is less, or where some other attraction is opposed to the agency of that element. When we consider that the urine of graminivorous animals contains a large quantity of free alkali, which is secreted from the blood; that, consequently, in the blood a current of dissolved alkalies is carried through the whole mass of the body, and especially through the substance of the muscles, while the fluid which is in contact with the external part of the blood-vessels and lymphatics (the juice of flesh) retains an acid reaction, we perceive that a cause must necessarily be in action at these points, which prevents the removal of the free acids, or, if they are removed, reproduces them at each moment of time.

"The blood-vessels and lymphatics contain an alkaline fluid, while the surrounding fluid, that of the flesh, is acid; the tissue of which the vessels are composed is permeable for the one or the other of these fluids. Here then are two conditions favorable to the production of an electrical current, and it is far from improbable, that such a current takes a certain share in the vital processes, although its action be not always indicated by proper electrical effects." P. 104.

"From the great difference of chemical nature and qualities in the fluids circulating in the different parts of the organism, it follows, that there must be a very remarkable difference in the permeability of the parietes of the vessels for these fluids. Were this permeability in all cases the same, there must have been found as much of the salts of soda and potash in the juice of flesh as in the blood; but the blood of the ox and the fowl contains nearly a third of its whole saline contents of chloride of sodium, while hardly a trace of this compound occurs in the juice of flesh.

"The vessels which secrete milk must stand in a similar relation to the blood-vessels; for in the milk of the cow, the salts of potash preponderate very greatly over those of soda, and are present also in much larger quantity than in the saline constituents of blood.

"In some pathological conditions there has been observed, at points where bones and muscles meet, an accumulation of free lactic and phosphoric acids, which has never been perceived at those points in the normal state. The solution and removal of the phosphate of lime, and therefore the disappearance of the bones, is a consequence of this state. It is not improbable that the cause, or one of the causes, of this separation of acid from the substance of the muscle, is this—that the vessels, which contain the fluid of the muscles, have undergone a change, whereby they lose the property of retaining within them the acid fluid they contain.

"The constant occurrence of chloride of sodium and phosphate of soda in the blood, and that of phosphate of potash and chloride of potassium in the juice of flesh, justifies the assumption that both facts are altogether indispensable for the processes carried on in the blood and in the fluid of the muscles.

" Proceeding on this assumption, the necessity for adding common salt to the food of many animals is easily explained, as well as the share which that salt takes in the formation of blood, and in the respiratory process." P. 109.

*Practical Applications.*—In this section Professor Liebig furnishes some useful hints upon the modes in which animal substances can be most advantageously prepared for human sustenance. From raw flesh intended to reproduce itself in the animal frame, none of its constituents should be withdrawn during the progress of cooking. " The water in which flesh has been boiled contains soluble alkaline phosphates, lactates, and isoninates, phosphate of magnesia, and only traces of phosphate of lime; the boiled flesh contains chiefly, with the fibrine, &c., the insoluble inorganic constituents, phosphate of lime and phosphate of magnesia." If therefore, the flesh be eaten without the broth it becomes the less adapted for nutrition, and that in proportion to the quantity of water employed, and the duration of the boiling. " It is clear that all the sapid and odorous constituents of flesh exist in the flesh itself in the soluble state, and consequently when it is boiled are transferred to the soup. The smell and taste of roasted flesh arise from the soluble constituents of the juice, which have undergone a slight change under the influence of the higher temperature. Flesh, which has been rendered quite tasteless by boiling with water, acquires the taste and all the peculiarities of roasted flesh, when it is moistened and warmed with a cold aqueous infusion of raw flesh, which has been evaporated until it has acquired a dark brown colour." It is upon those soluble constituents the peculiar flavour of each kind of flesh depends, so that if to boiled beef a concentrated cold aqueous infusion of vension or fowl be added, and the mass warmed, the beef can no longer be distinguished from venison or fowl. " A slight addition of lactic acid (a very little sauer kraut, for example), or of chloride of potassium, which is an invariable constituent of all infusions of flesh, heightens the piquancy of the flavour of meat; as, on the other hand, an alkaline liquid, or the addition of blood, renders the soup of meat utterly insipid and mawkish." By the agency of cold water, also, all the *albumen* which every where surrounds the muscular fibre in a liquid form may be obtained; the quantity obtainable bearing an inverse ratio to the age of the animal. Old animals are, however, richest in fibrine, which, when deprived of its albumen becomes hard and horny in proportion as it is boiled. The tenderness of cooked meat, therefore, depends upon the amount of albumen coagulating between the fibres, and thus preventing the hardening of these. The duration of the boiling is another point to be attended to; for when this is too prolonged the albumen becomes harder, though not tough.

The following are the directions given for the best method of *boiling meat.*

" If the flesh intended to be eaten be introduced into the boiler when the water is in a state of brisk ebullition, and if the boiling be kept up for some minutes then so much cold water added as to reduce the temperature of the water to 165° or 158°, and the whole kept at this temperature for some hours, all the conditions are united, which give to the flesh the quality best adapted to its use as food.

"When it is introduced into the boiling water, the albumen immediately coagulates from the surface inwards, and in this state forms a crust or shell, which no longer permits the external water to penetrate into the interior of the mass of flesh. But the temperature is gradually transmitted to the interior, and there effects the conversion of the raw flesh into the state of boiled or roasted meat. The flesh retains its juiciness, and is quite as agreeable to the taste as it can be made by roasting; for the chief part of the sapid constituents of the mass is retained under these circumstances, in the flesh.

"If we reflect that the albumen of the juice of flesh begins to coagulate at a temperature of 105·5° and that it is completely coagulated at 140° (Berzelius), it might be supposed that it would not be necessary, in the cooking of flesh, to expose it to a higher temperature than 140°. But, at that temperature, the colouring matter of the blood is not yet coagulated; the flesh, indeed, is eatable, but when it contains blood, it acquires, under these circumstances, a bloody appearance, which it only loses, when it has acquired, throughout the whole mass, a temperature of 150° to 158°.

"The introduction of the piece of raw flesh into water already boiling is the best process for the dressing of the meat, but the most unfavourable for the quality of the soup. If, on the contrary, the piece of raw meat be placed in cold water, and this brought *very gradually* to the boiling point, there occurs, from the first moment, an interchange between the juices of the flesh and the external water. The soluble and sapid constituents of the flesh are dissolved in the water, and the water penetrates into the interior of the mass, which it extracts more or less completely. The flesh loses, while the soup gains in sapid matters; and, by the separation of albumen, which is commonly removed by skimming, as it rises to the surface of the water when coagulated, the surface of the meat more particularly loses its tenderness and shortness (as it is called), becoming tough and hard. The thinner the piece of flesh, the more completely does it acquire the last-mentioned qualities; and if in this state it be eaten without the soup, it not only loses much of its nutritive properties, but also of its digestibility, inasmuch as the juice of the flesh itself, the constituents of which are now found in the soup, are thus prevented from taking part in the digestive process in the stomach. The soup, in fact, contains two of the chief constituents of the gastric juice." P. 128.

It has long been supposed that the chief properties of soup depended upon the amount of gelatinous matter dissolved during boiling; but, in fact, gelatine is tasteless and exists in a very small quantity in properly prepared soup. The nutritious and sapid ingredients exist ready formed in the flesh, and are in no-wise the products of the boiling. They may be most promptly extracted by following this formula for the preparation of soup:—

"When 1 lb. of lean beef, free of fat, and separated from the bones, in the finely-chopped state in which it is used for beef-sausages or mince-meat, is uniformly mixed with its own weight of cold water, slowly heated to boiling, and the liquid, after boiling briskly for a minute or two, is strained through a towel from the coagulated albumen and the fibrine, now become hard and horny, we obtain an equal weight of the most aromatic soup, of such strength as cannot be obtained, even by boiling for hours, from a piece of flesh. When mixed with salt and the other usual additions by which soup is usually seasoned, and tinged somewhat darker by means of roasted onions or burnt sugar, it forms the very best soup which can in any way be prepared from 1 lb. of flesh." P. 132.

By concentration and slow evaporation a valuable extract of flesh may be prepared, half-an-ounce of which converts 1 lb. of water into a strong well-flavoured soup. This portable soup (a very different one from that ordinarily so called which consists of gelatine), although too dear to form an article of commerce, is well worthy the attention of governments, as a restorative for wounded soldiers, and in the provisioning of ships and fortresses, as a means of preventing or relieving disease arising from a dearth of fresh meat and vegetables.

The effects of *salting meat* have been carefully investigated by Liebig, and he has ascertained that the brine in contact with the meat contains the constituents of a concentrated soup or infusion of meat; so that by this process the composition of meat becomes much more changed than by boiling. In the latter, " the highly nutritious albumen remains in the coagulated state in the mass of flesh, but, in salting, the albumen is separated from the flesh; for when the brine from salt meat is heated to boiling, a large quantity of albumen separates as a coagulum." It contains likewise, lactic acid, the phosphates, kreatine and kreatinine; and, in proportion as these are abstracted from the meat does it lose its nutritive properties. A portion of such flesh is converted into an element of respiration; and health cannot be maintained upon salt meat, unless a much larger quantity be given, " inasmuch as it cannot perfectly replace, by the substances it contains, those parts of the body which have been expelled in consequence of the change of matter, nor can it preserve in its normal state the fluid distributed in every part of the body, namely, the juices of flesh."

We conclude our notice of Professor Liebig's interesting work with the following extract:—

" If we consider that the juice of flesh, in all animals yet examined, possesses a constant character; that, exclusive of those constituents which are derived from the blood unavoidably mixed with it, as well as of small quantities of odorous and sapid substances on which the characteristic secondary or by-taste of the juice or soup of the flesh in each kind of animal depends, the juice of ox-flesh is in no way distinguishable from that of the fox, it seems justifiable to conclude, that the quantity and the nature of the soluble constituents in the muscular system are essential to the functions of the muscles. It appears further to follow, that in judging of the nutritive qualities of any kind of food, the composition of the blood cannot be selected as the proper datum from which to argue, because there are a number of factors which must be brought into the calculation, and which are either wanting in the blood, or present in it only in trifling quantity.

" Some experiments have lately been made by Lehmann on the gastric juice of dogs, fed on bones and lean horse-flesh, which fluid he has studied more minutely than had previously been done. He obtained from it a crystallised salt of magnesia, combined with an organic acid, not containing nitrogen. This salt yielded 16.6 p. c. of magnesia, and 21 p. c. of water of crystallisation. Now that we know that lactic acid forms a constituent of the chief mass of the body, it is evident that Lehmann's magnesian salt, which agrees with lactate of magnesia in the proportion of base and of water of crystallisation, really was lactate of magnesia. In that case the gastric juice contains lactic acid, and thus the problem of the digestive process in the stomach would appear, in its chemical aspect, to be completely solved.

" The experiments of all who have studied the gastric juice agree in this, that that fluid contains, along with an organic acid, free phosphoric acid or an acid phosphate, and in this respect its similarity with the juice of the muscles is strikingly obvious. That portion of the gastric juice which is soluble in alcohol is, in its reaction, identical with the alcoholic extract of soup, as Tiedemann and Gmelin have already shown; and the soup or infusion of meat, free from gelatine and fat, the preparation of which I have described, may perhaps admit of being employed as a valuable remedy for many dyspeptic patients, with the view of increasing the activity of the stomach, and promoting digestion. Again, if the blood or the muscular substance of emaciated convalescents cannot supply the matters necessary for digestion in sufficient quantity for a rapid reproduction of the lost strength (that is, the lost parts of the organism), the benefit derived from well-made soup during convalescence admits of a simple explanation." P. 139.

Although forming but a single chapter in continuation of his work upon the "Chemistry of Vegetable and Animal Physiology," Professor Mulder, as Mr. Johnston remarks, has in fact produced a distinct treatise upon a novel subject—CHEMICO-HISTOLOGY. "The anatomical doctrine of minute structures can only be investigated by the skilful use of the microscope. Their chemical nature, and the changes of composition which they undergo during progressive growth, or by the agency of disease, can only be determined by the joint use of the microscope and of varied chemical re-agents. The skill and dexterity of the ordinary histologist must be united to the knowledge and refined manipulations of the practised chemist, before any results can be obtained, which shall be of real service to vegetable and animal physiology." In this point of view the present work is not merely the best but the only one of the kind existing in which the student will find the histological and chemical characteristics of the various vegetable and animal tissues set forth with remarkable perspicuity and completeness, and illustrated by some excellent coloured engravings. Whatever may eventually be the fate of the author's views concerning proteine and its oxides, these researches and observations must ever remain a monument to his analytical skill and unwearied industry. The instructive details in which this work abounds must be referred to in the book itself, which will become an indispensable companion to every chemist and microscopist, who is desirous of acquiring a correct knowledge of structures hitherto imperfectly described or erroneously confounded with each other. One or two of the more general observations are all we can attempt to lay before our readers. Treating of the importance of considering the form, as well as the composition of parts, he observes:—

"The invaluable investigations, which of late years have been made with the microscope, have proved, that organs performing the same functions consist of the same kind of tissue; whilst those performing different functions consist of different tissues. This leads to the just conclusion, that the form of the parts of which an organ is built up, or by which a function is performed, is in close connection with that function itself, and that, on the other hand, the function is dependent upon the form. If the form be really dependent on matter and the forces residing therein, the function is dependent upon this also. In organic nature, therefore, matter, form, and function go together, and the one being different, the other will necessarily be different also.

"This view of the subject applies as well to all the sickly conditions of any organ, or of the whole organism, as it does to all the healthy functions of plants and animals. The connection existing between different organs of the animal body by nerves and vessels, and both of the animal and vegetable body by cells, is of such a kind, that Bichat's wish—that the organism might some day be made divisible into such simple parts, that conceptions of vital action might be attached to them, equally simple as those of gravity, elasticity, &c., connected with unorganised bodies,—will probably never be realised. But although these conceptions will never attain the greatest possible simplicity, as regards the connection of the constituent parts in an organic whole; we can, nevertheless, approach to that simplicity, and we do approach to it now that we have commenced to establish a connection between form, matter, and function.

"For these reasons a brief survey of the elementary forms of organised parts is at present peculiarly suitable. Where we have as yet but insufficient knowledge of the mutual connection of form, matter, and function, which is too often the case, it will be useful to become acquainted with the forms, though it be only as a part of the chemical history of the organic body. The chemist ought to see something more than gelatine in gelatinous tissue, and the histologist something more than fibres of a determined form. It appears to me, that it is equally necessary for the chemist to know the elementary forms of the tissues, as to know that alum crystallises as an octohedron. It is chiefly to the investigations of Schleiden and of Schwann that we are indebted for our knowledge of these elementary forms." P. 353.

There then follows a concise and excellent account of the Cell-theory as developed by those enquirers, as well as an appreciation of the various objections and modifications that have been subsequently advanced. An extract or two illustrative of this subject may be given.

"But whatever conception we may have of the cell-formation, it is entirely undeniable, 1° That, as there are soluble inorganic substances, which in certain conditions crystallize, so there are soluble organic substances, which in certain conditions form nucleoli, kernels, and cells. 2° That these little organs maintain themselves, and preserve their specific characters, as individuals do by generation, according to the universal law of organic nature, that, the conditions being the same, like produces like. 3° That the endogenous formation of cells is the result of the conversion of organic substances into such as are similar to those of the generating cell itself. The substance must first be made similar to that which has a form, before it can assume that form. 4° That the generating cell, therefore, has but one function to perform—the preparation of a substance, identical with that of which itself consists. This substance being once prepared, its organisation is as inevitable a consequence, as crystallisation is a consequence of the mixing of nitric acid and baryta water,—of sulphuric acid and potash, &c. 5° That the parent cell, therefore, does not give a form to the young cells, any more than a mother gives a form to her child. A generating being does nothing more than secrete a substance, which, under certain circumstances, can assume a form similar to that of its parent. In this respect there is no difference whatever between the propagation of the higher animals, and the multiplication of cells." P. 367.

Upon the differences between the vegetable and animal cells, Dr. Mulder thus remarks:—

"The cells of plants present some points of similarity with those of animals, but at the same time they are characterized by important differences. The most essential point of similarity is, undoubtedly, the independence of their existence and action.

"But the differences between vegetable and animal cells are as great as the difference of the orders of beings to which they respectively belong. A vegetable cell cannot produce an animal tissue, neither can an animal cell give rise to a vegetable one. The substances from which the former cells are built up, are entirely different from those of the latter. In not one animal cell has even a trace ever been found of that substance which constitutes the whole of an unincrusted vegetable cell, viz. cellulose. The latter being in all plants, without exception, the material of the original cells, is the cause of the similarity in the structure and functions of plants; and its absence in animals is the cause of the difference between vegetable and animal cells. However cells may be changed by age, the cellulose never entirely disappears, though other substances are often deposited upon it.

"Another remarkable difference between the cells of plants and those of animals is, that the former have a great tendency to preserve—the latter to lose—the cellular form. With the exception of the different kinds of epithelium, feathers, cartilaginous tissue, and a few other substances, the animal tissues, when full grown, always consist of transformed cells or other original particles, produced from cell kernels. These particles have often retained nothing of what they were during their development, except the cell kernel or nucleus, and sometimes not even this; whilst, on the other hand, the great mass of plants, both vascular and cellular, continues to consist of real cells, of which only the walls increase in thickness. Even those parts which are called vessels by botanists,—with the exception of the laticiferous and the spiral vessels,—are nothing but a kind of elongated cells, and to them, therefore, the name of cells is equally applicable.

"There still rests, alas! a thick cloud over our knowledge of the cells of plants, although the most acute philosophers have, with the greatest exertion, directed their attention to the subject. Different cellular systems, though similar in form, though apparently of the same chemical composition, though drawing their food from the same source, produce very different substances. In general the greatest conformity prevails in regard to the cells of plants, notwithstanding the variety in the form and substance of their products. One universal substance, cellulose, exists in *all* plants, constituting the basis, the material of their cells. But this very fact, that one single substance can assume the most different forms, prevents us as yet from giving any satisfactory exposition of this part of natural science." P. 399.

